# Effect of Continuity of Care Combined With Family Supportive Care on Cognitive Function and Self-Care Ability in Patients With Alzheimer's Disease

**DOI:** 10.62641/aep.v54i1.2094

**Published:** 2026-02-15

**Authors:** Min Wang, Yonghui Xu, Huina Wang, Shurui Dou, Jingjing Xu, Fanfan Wang, Yan Xu

**Affiliations:** ^1^Department of Cardiology, China-Japan Friendship Hospital, 100038 Beijing, China; ^2^Department of Neurology, China-Japan Friendship Hospital, 100038 Beijing, China

**Keywords:** Alzheimer's disease, continuity of patient care, cognition

## Abstract

**Background:**

This study aimed to investigate the effects of a combined approach of continuity of care and family supportive care on cognitive function and self-care ability in patients with Alzheimer's disease (AD).

**Methods:**

The clinical data of 135 patients with AD, who presented to China-Japan Friendship Hospital from April 2021 to April 2023, were retrospectively analysed. On the basis of the sequential introduction of different care protocols at China-Japan Friendship Hospital, the patients were categorised into three groups: the conventional group (n = 42, receiving conventional care), the continuity group (n = 49, receiving conventional care plus continuity of care) and the family group (n = 44, receiving conventional care, continuity of care, and family supportive care). Cognitive function (assessed using the Mini-Mental State Examination and Montreal Cognitive Assessment), self-care ability measured using the Barthel Index (BI), family support evaluated using the Perceived Social Support from Family Scale (PSS-Fa), and quality of life assessed via the Quality of Life in Alzheimer's Disease scale (QOL-AD) were compared across the three groups before and 3 months after the implementation of the respective care protocols.

**Results:**

At the 3-month mark, the family group demonstrated significantly higher BI, PSS-Fa and QOL-AD scores than the continuity and conventional groups, and the continuity group's scores on these measures were significantly higher than those of the conventional group (*p* < 0.05).

**Conclusions:**

The application of continuity of care combined with family supportive care in patients with AD is associated with positive effects on cognitive function, self-care ability, family support and quality of life. This finding suggests that this integrated care model may represent a superior option for the management of patients with AD. However, given the inherent limitations of retrospective designs in fully controlling for confounding variables, further validation through prospective, large-sample studies is warranted to confirm its efficacy and generalisability.

## Introduction

Alzheimer’s disease (AD) is a progressive neurodegenerative disorder predominantly occurring in presenile and senile periods, characterised by cognitive decline, behavioural changes and functional impairment, which not only severely diminishes patients’ quality of life but also imposes a substantial burden on families and society [1]. Currently, no curative treatment is available for AD, and clinical management primarily relies on pharmacological agents, such as cholinesterase inhibitors (ChEIs) and N-methyl-D-aspartate (NMDA) receptor antagonists, to delay disease progression [2, 3]. However, pharmacotherapy alone often yields suboptimal outcomes. The implementation of additional healthcare and support measures is necessitated to enhance overall therapeutic efficacy; improve cognitive function to the greatest extent possible; and alleviate neuropsychiatric symptoms (NPSs) such as apathy, anxiety and agitation. The 2018 guidelines on risk factors and management of AD and the 2019 Chinese expert consensus on the rehabilitation management of AD explicitly emphasise that integrating pharmacotherapy with nursing care is a pivotal direction in AD rehabilitation management [4, 5].

Conventional care, primarily relying on pre-discharge rehabilitation guidance and health instruction from primary healthcare institutions during home residence, often remains at a basic level, such as medication reminders, exhibiting relatively insufficient professional rigor and failing to provide personalised care. Continuity of care refers to comprehensive, systematic and continuous nursing services provided by professional nurses to patients after discharge, including health education, rehabilitation guidance and psychological support, which can enhance the quality of care to a certain extent [6, 7]. Influenced by traditional cultural practices and other factors, the proportion of patients with AD in China receiving professional nursing within medical institutions is relatively low. The family serves as the primary setting for daily living and rehabilitation for these patients, who rely heavily on long-term family care. Sole reliance on continuity of care fails to adequately incorporate and leverage the indispensable role of family caregivers, indicating room for further improvement in care quality. Family supportive care denotes a nursing model wherein family members provide essential medical, rehabilitative and psychological support services to patients within the home environment, which is conducive to enhancing patients’ self-management abilities and life satisfaction [8]. Previous studies have confirmed that family supportive care can optimise asthma control in children with asthma and improve self-management capacity and quality of life in patients with acute cerebral infarction [9, 10]. Furthermore, a randomised controlled trial conducted by Boltz *et al*. [11] demonstrated that family-centred care protocols can improve the functional status and quality of care for patients with AD. Hovenga *et al*. [12] indicated that family involvement in care contributes to an enhanced quality of life for individuals with AD. Theoretically, integrating family supportive care with continuity of care could enhance overall nursing quality; however, reports on the combined application of these two approaches remain scarce. The present study was designed to investigate the effects of combining continuity of care with family supportive care on cognitive function and self-care ability in patients with AD. The details are reported as follows.

## Materials and Methods

### General Information

This study was conducted in accordance with the ethical principles of the World Medical Association Declaration of Helsinki [13]. Informed consent was obtained from all patients and their family members. The study protocol was reviewed and approved by the Hospital Ethics Committee (Approval No. 2023011021). In the initial screening, 142 patients with AD who met the inclusion criteria were identified from all those attending China-Japan Friendship Hospital between April 2021 and April 2023. After one patient with a history of intracranial surgery, two patients with comorbid chronic heart failure and four patients clinically diagnosed with moderate to severe cognitive impairment were excluded, 135 patients with AD were ultimately enrolled in the study. The patients were categorised into three groups on the basis of the sequential introduction of different care protocols at China-Japan Friendship Hospital: the conventional group (n = 42), which included patients who received conventional care from April 2021 to October 2021; the continuity group (n = 49), comprising patients who received conventional care plus continuity of care from November 2021 to July 2022; and the family group (n = 44), consisting of patients who received conventional care, continuity of care and family supportive care from August 2022 to April 2023.

### Inclusion and Exclusion Criteria

Inclusion criteria: (a) met the diagnostic criteria for AD as established by the National Institute on Aging and the Alzheimer’s Association or the Alzheimer’s Disease Chinese [14, 15, 16]; (b) exhibited stable vital signs and NPSs; (c) had no immune system diseases or coagulation dysfunction; (d) possessed basic language communication, auditory, visual and comprehension abilities, enabling cooperation with relevant assessments; (e) had no severe physical comorbidities; (f) were in the mild dementia stage, assessed as Global Deterioration Scale (GDS) stages 2–3 [17]; (g) had a family member or other relative willing to participate in family care and serving as the primary caregiver; and (h) had complete clinical data.

Exclusion criteria: (a) history of psychotropic drug abuse; (b) other types of dementia or cognitive impairment not caused by AD; (c) comorbid other neurological diseases or a clinical diagnosis of moderate to severe cognitive impairment; (d) comorbid severe dysfunction of major organs (e.g., heart, lung, kidney and liver); (e) withdrew from the study midway or experienced major life events within the family during the study period; (f) comorbid malignant tumours, haematological diseases or infectious diseases; (g) history of intracranial surgery or use of antidepressants or other psychotropic medications within the past month; (h) the completion rate of the assigned care protocol falling below 85% during the study period for any reason (e.g., disease progression, caregiver time constraints, change in residence and major family life events). The completion rate was calculated as follows: (actual number of protocol sessions delivered/total number of planned sessions) × 100%.

### Methods

#### Conventional Group

The patients in this group received conventional care. Prior to discharge, health education pamphlets were distributed by nursing staff, and standard health education was provided to the patients and their families, emphasising the importance of adherence to medication and appropriate physical exercise. Following discharge, regular telephone follow-ups were conducted to monitor medication compliance, disease progression and rehabilitation status, during which necessary guidance and recommendations were provided. This care regimen was maintained for a total of 3 months.

#### Continuity Group

This group received continuity of care in addition to the conventional care protocol. Prior to discharge, the patient’s clinical condition was assessed to formulate an individualised home-based rehabilitation plan. This plan encompassed dietary management, language function training, motor exercise and activities-of-daily-living (ADL) training. Following discharge, a combined approach was implemented utilising WeChat video calls (once weekly, lasting approximately 20–30 min per session) complemented by home visits (once monthly). Through these modalities, guided instruction was provided on cognitive training, physical exercise, speech practice and skills to enhance daily living capabilities. This protocol was sustained for a period of 3 months. Specific cognitive training activities included memory exercises (e.g., guiding patients to recall 3–5 items or their home address) and attention training (e.g., sequencing numbers, progressively increasing from 3- to 5-digit sequences).

#### Family Group

This group received family supportive care integrated with the continuity of care protocol provided to the continuity group. As outlined in the introduction, family supportive care is defined as a nursing model wherein family members provide medical, rehabilitative and psychological support services within the home environment; all specific components of this protocol were designed around this core definition. Prior to discharge, multimedia resources, such as PowerPoint presentations and short videos, were utilised to educate caregivers on the significance and key elements of family supportive care. Caregivers were assisted in formulating detailed, individualised home care plans. Demonstrations of daily care techniques were provided, including guided feeding (using brightly coloured utensils to attract the patient’s attention) and progressive bathing assistance (initially assisting with undressing then guiding the patient to perform self-wiping). Role-playing scenarios were conducted to simulate situations where patients resisted care, allowing caregivers to practice appropriate responses. Training modules were designed around the themes of cognitive impairment, communication skills, ADL, physical exercise and psychological support, tailored to the patient’s cultural background and lifestyle. Cognitive training involved daily activities progressing from simple to complex, such as conversations, storytelling and reminiscence therapy (with a unified nostalgic theme focusing on the patient’s young adulthood, considerable family anniversaries or stories of familiar relatives/friends; conducted once daily for 20 min per session, guided by caregivers using pre-set topics), organised newspaper reading, number sequencing, paper folding, card games, recalling three daily objects and reciting home addresses. Communication training was integrated into daily dialogues; caregivers were instructed to avoid arguments; refrain from asking more than two questions simultaneously; and supplement verbal communication with gestures, smiles, and eye contact. Positive feedback and encouragement were consistently provided. If the patient exhibited frustration, the caregivers were coached to wait patiently for a response before offering suggestions. For ADL training, the patients were encouraged to independently perform daily activities (e.g., dressing, face washing, tooth brushing and toileting) to the greatest extent possible. The caregivers were guided in maintaining the patient’s personal hygiene through regular nail trimming, shaving, haircuts and appropriate grooming. The physical exercise regimen included daily walks on flat, familiar routes, supplemented by balance training (e.g., single-leg standing, toe walking and Tai Chi), flexibility exercises (e.g., shoulder rotations, wrist/ankle circles and dancing) and strength training (e.g., seated leg lifts) tailored to the patient’s capabilities. Psychological support involved three fixed 15 min of daily music listening sessions (scheduled 30 min after breakfast, 1 h before noon rest and 1 h before bedtime). Music selection comprised familiar, nostalgic tunes (e.g., revolutionary songs and classic folk songs) or soft, relaxing music (e.g., universally recognised pieces like Beethoven’s ‘Moonlight Sonata’), excluding pieces with fast tempos or melodies that are full of passion. During these sessions, the patients were guided to perform simple motor activities like clapping or stepping in rhythm with the music. A dedicated WeChat communication group involving healthcare professionals was established. The caregivers were encouraged to share daily challenges and experiences within this platform, and the nurses provided daily summaries and feedback. This comprehensive protocol was sustained for a period of 3 months.

#### Protocol Completion Rate Across Groups

The follow-up records of patients and communication logs of caregivers across the three groups were reviewed to compare their protocol completion rates. After screening was conducted on the basis of the inclusion and exclusion criteria, the enrolled patients in all three groups demonstrated good adherence to the assigned protocols. The mean protocol completion rate was 98.27% ± 3.06% for the conventional group, 99.15% ± 3.43% for the continuity group and 99.38% ± 3.57% for the family group. Comparison of the completion rates amongst the three groups revealed no statistically significant difference (*F *= 1.302, *p* > 0.05).

### Outcome Measures

(1) Cognitive function: Cognitive function was evaluated by comparing the scores on the Chinese version of the Mini-Mental State Examination (MMSE) and the Chinese version of the Montreal Cognitive Assessment (MoCA) before and 3 months after the implementation of the care protocols across the three groups. The MMSE, originally developed by Folstein *et al*. [18] and translated/validated in Chinese by Jia *et al*. [19], assesses cognitive domains including attention and orientation, with total scores ranging from 0 to 30; higher scores indicate better cognitive function. The MoCA, developed by Nasreddine *et al*. [20] and translated/validated in Chinese by Zhang and Liu [21], assesses domains such as visuospatial/executive function and delayed recall, with total scores ranging from 0 to 30; higher scores denote superior cognitive performance. In the present study, the Cronbach’s α coefficients for MMSE and MoCA were 0.89 and 0.87, respectively.

(2) Self-care ability: Self-care ability was assessed by comparing the scores on the Chinese version of the Barthel Index (BI) before and 3 months after the implementation of the care protocols across the three groups. The BI, originally developed by Mahoney and Barthel [22] and translated/modified in Chinese by Min *et al*. [23], encompasses 10 items, including feeding and dressing, with total scores ranging from 0 to 100; higher scores reflect greater independence in ADL. The Cronbach’s α coefficient for the BI in the present study was 0.87.

(3) Family support and quality of life: Family support and quality of life were evaluated by comparing the scores on the Chinese version of the Perceived Social Support from Family Scale (PSS-Fa) and the Chinese version of the Quality of Life in Alzheimer’s Disease scale (QOL-AD) before and 3 months after the implementation of the care protocols across the three groups. PSS-Fa, developed by Procidano and Heller [24] and translated into Chinese by Zhang and Liu [25], consists of 15 items scored on a 0 or 1 point scale (yes = 1, no = 0), with total scores ranging from 0 to 15; higher scores indicate greater perceived family support. QOL-AD, developed by Logsdon *et al*. [26, 27] and translated/validated in Chinese by Zhang *et al*. [28], comprises 13 items rated on a 4-point Likert scale (poor = 1, fair = 2, good = 3 and excellent = 4), yielding total scores between 13 and 52; higher scores signify better quality of life. In the present study, the Cronbach’s α coefficients for PSS-Fa and QOL-AD were 0.88 and 0.85, respectively.

### Statistical Analysis

Data analysis was performed using SPSS software (version 27.0; IBM Corp., Armonk, NY, USA). The normality of data distribution was first assessed using Shapiro–Wilk test. Continuous variables conforming to a normal distribution are presented as mean (±standard deviation), whereas non-normally distributed data are expressed as median (interquartile range) [M (Q1, Q3)]. For between-group comparisons of normally distributed continuous data, independent sample *t*-test was employed; otherwise, Mann–Whitney U test was used. For within-group comparisons of normally distributed continuous data, paired sample *t*-test was applied; alternatively, Wilcoxon signed-rank test was used. Categorical data are presented as n (%), and comparisons between and within groups were conducted using Chi-square test or Fisher’s exact probability test, as appropriate.

Given the significant potential confounding effect of educational attainment on MMSE and MoCA scores, a stratified analysis was performed to control for this confounder. The participants were stratified by educational level (stratum 1: illiterate; stratum 2: primary/junior high school; stratum 3: high school and above). One-way analysis of variance (ANOVA) was used for comparisons amongst multiple groups, with LSD-t test applied for post-hoc pairwise comparisons. A *p*-value < 0.05 was considered statistically significant.

Interaction effect analysis (educational stratum × care group) was conducted to verify the consistency of the care effects across strata. A *p*-value > 0.05 for the interaction term indicated no significant difference in the cognitive improvement effect of the care protocols amongst patients with different educational backgrounds, suggesting consistent results across strata.

## Results

### Baseline Characteristics

The comparison of baseline characteristics demonstrated good comparability amongst the three groups (*p* > 0.05, Table 1).

**Table 1.** **Comparison of baseline characteristics amongst three groups**.

Characteristic		Conventional group (n = 42)	Continuity group (n = 49)	Family group (n = 44)	χ^2^/*F*	*p*
Age (Years)		71.38 ± 4.36	72.06 ± 4.45	72.17 ± 4.82	*F *= 0.382	0.683
Gender						
	Male	2 (4.76)	3 (6.12)	2 (4.55)	χ^2^ = 0.140	0.933
	Female	40 (95.24)	46 (93.88)	42 (95.45)		
Disease duration (years)		2.81 ± 0.25	2.86 ± 0.27	2.91 ± 0.28	*F *= 1.504	0.226
BMI (kg/m^2^)		22.03 ± 1.12	22.39 ± 1.24	22.47 ± 1.36	*F *= 1.524	0.222
Medication						
	ChEIs	39 (92.86)	45 (91.84)	42 (95.45)	χ^2^ = 0.510	0.775
	ChEIs + NMDA Receptor Antagonist	3 (7.14)	4 (8.16)	2 (4.55)		
Educational level						
	Illiterate	13 (30.95)	16 (32.65)	11 (25.00)	χ^2^ = 1.130	0.890
	Primary/Junior High School	20 (47.62)	25 (51.02)	23 (52.27)		
	High School and Above	9 (21.43)	8 (16.33)	10 (22.73)		
AD type						
	EOAD	4 (9.52)	5 (10.20)	3 (6.82)	χ^2^ = 0.359	0.836
	LOAD	38 (90.48)	44 (89.80)	41 (93.18)		
GDS stage						
	Stage 2	30 (71.43)	33 (67.35)	34 (77.27)	χ^2^ = 1.135	0.567
	Stage 3	12 (28.57)	16 (32.65)	10 (22.73)		
Primary caregiver age (Years)		46.43 ± 3.32	47.15 ± 3.48	46.96 ± 3.51	*F *= 0.520	0.596
Primary caregiver education						
	Illiterate	5 (11.90)	3 (6.12)	6 (13.64)	χ^2^ = 2.190	0.701
	Primary/Junior High School	18 (42.86)	20 (40.82)	15 (34.09)		
	High School and Above	19 (45.42)	26 (53.06)	23 (52.27)		
Primary caregiver gender						
	Male	17 (40.48)	19 (38.78)	16 (36.36)	χ^2^ = 0.156	0.925
	Female	25 (59.52)	30 (61.22)	28 (63.64)		
Hypertension						
	Yes	8 (19.05)	10 (20.41)	8 (18.18)	χ^2^ = 0.076	0.963
	No	34 (80.95)	39 (79.59)	36 (81.82)		
Diabetes mellitus						
	Yes	3 (7.14)	4 (8.16)	2 (4.55)	χ^2^ = 0.510	0.775
	No	39 (92.86)	45 (91.84)	42 (95.45)		

Note: BMI, Body Mass Index; ChEIs, Cholinesterase Inhibitors; NMDA, N-Methyl-D-Aspartate; AD, Alzheimer’s disease; EOAD, Early-Onset Alzheimer’s Disease; LOAD, Late-Onset Alzheimer’s Disease; GDS, Global Deterioration Scale.

### Cognitive Function 

Compared with baseline measurements within each group, the MMSE and MoCA scores were significantly increased in all three groups at the 3-month assessment (*p* < 0.05). At the 3-month mark, the family group demonstrated significantly higher MMSE and MoCA scores than the continuity and conventional groups, and the continuity group’s scores on these measures were significantly higher than those of the conventional group (*p* < 0.05, Table 2). Within each educational attainment stratum, the MMSE and MoCA scores at 3 months significantly improved compared with baseline in all three groups (*p* < 0.05). Furthermore, within each stratum, the scores followed a consistent pattern, namely, family group > continuity group > conventional group, with pairwise comparisons revealing statistically significant differences (*p* < 0.05). Analysis of interaction effects indicated that the *p*-values for the ‘educational stratum × care group’ interaction were 0.723 for MMSE and 0.689 for MoCA (*p* > 0.05, Table 3), suggesting that the cognitive function improvement associated with the care protocols was consistent across patients with different educational backgrounds.

**Table 2.** **Comparison of MMSE and MoCA scores before and 3 months after implementation of care protocols amongst three groups (points)**.

Group	MMSE score		MoCA score	
	Before implementation	Three months after implementation	Before implementation	Three months after implementation
Conventional group (n = 42)	21.04 ± 1.16	22.31 ± 1.82*	20.86 ± 1.23	22.45 ± 1.87*
Continuity group (n = 49)	21.35 ± 1.21	23.79 ± 1.93*^a^	21.05 ± 1.34	24.13 ± 1.98*^a^
Family group (n = 44)	21.17 ± 1.09	25.26 ± 2.07*^ab^	20.76 ± 1.18	25.81 ± 2.15*^ab^
*F*	0.828	24.745	0.644	30.198
*p*	0.439	<0.001	0.527	<0.001

Note: *p < 0.05 versus before implementation within the same group; ^a^*p* < 0.05 versus conventional group; ^b^*p* < 0.05 versus continuity group. MMSE, Mini-Mental State Examination; MoCA, Montreal Cognitive Assessment.

**Table 3.** **Comparison of MMSE and MoCA scores before and 3 months after implementation of care protocols, stratified by educational level (points)**.

Educational stratum	Group	MMSE score		MoCA score	
		Before implementation	Three months after implementation	Before implementation	Three months after implementation
Illiterate	Conventional group (n = 13)	19.21 ± 1.05	20.35 ± 1.12*	18.96 ± 1.08	20.12 ± 1.15*
	Continuity group (n = 16)	19.38 ± 1.02	21.87 ± 1.23*^a^	19.15 ± 1.03	22.05 ± 1.21*^a^
	Family group (n = 11)	19.15 ± 1.07	23.52 ± 1.31*^ab^	18.89 ± 1.05	23.78 ± 1.28*^ab^
	*F*	0.182	20.188	0.227	27.444
	*p*	0.835	<0.001	0.798	<0.001
Primary/Junior high school	Conventional group (n = 20)	21.35 ± 1.12	22.68 ± 1.25*	21.02 ± 1.15	22.89 ± 1.23*
	Continuity group (n = 25)	21.52 ± 1.08	23.95 ± 1.31*^a^	21.23 ± 1.11	24.32 ± 1.35*^a^
	Family group (n = 23)	21.28 ± 1.10	25.41 ± 1.38*^ab^	20.98 ± 1.09	25.96 ± 1.42*^ab^
	*F*	0.304	23.142	0.347	28.242
	*p*	0.739	<0.001	0.708	<0.001
High school and above	Conventional group (n = 9)	23.12 ± 1.23	24.51 ± 1.38*	22.89 ± 1.21	24.68 ± 1.35*
	Continuity group (n = 8)	23.32 ± 1.18	25.62 ± 1.45*^a^	23.05 ± 1.17	25.89 ± 1.42*^a^
	Family group (n = 10)	23.08 ± 1.21	26.89 ± 1.52*^ab^	22.76 ± 1.19	27.25 ± 1.48*^ab^
	*F*	0.097	6.372	0.132	7.787
	*p*	0.908	0.006	0.877	0.003
Interaction effect		*p* = 0.723		*p* = 0.689	

Note: *p < 0.05 versus before implementation within the same group; ^a^*p* < 0.05 versus conventional group; ^b^*p* < 0.05 versus continuity group. MMSE, Mini-Mental State Examination; MoCA, Montreal Cognitive Assessment. The interaction effect represents the test result for the ‘educational stratum × care group’ interaction term.

### Self-Care Ability

Compared with the baseline measurements within each group, the BI scores in all three groups significantly increased at the 3-month assessment (*p* < 0.05). At the 3-month mark, the family group demonstrated significantly higher BI scores than the continuity and conventional groups, and the BI scores in the continuity group were significantly higher than those in the conventional group (*p* < 0.05, Table 4).

**Table 4.** **Comparison of BI scores before and 3 months after implementation of care protocols amongst three groups (points)**.

Group	Before implementation	Three months after implementation
Conventional group (n = 42)	66.57 ± 4.33	73.71 ± 5.26*
Continuity group (n = 49)	67.08 ± 4.19	76.64 ± 5.48*^a^
Family group (n = 44)	65.96 ± 4.24	79.75 ± 6.13*^ab^
*F*	0.805	12.356
*p*	0.449	<0.001

Note: *p < 0.05 versus before implementation within the same group; ^a^*p* < 0.05 versus conventional group; ^b^*p* < 0.05 versus continuity group. BI, Barthel Index.

### Family Support and Quality of Life

Compared with the baseline measurements within each group, the scores on PSS-Fa and QOL-AD in all three groups significantly increased at the 3-month assessment (*p* < 0.05). At the 3-month mark, the family group demonstrated significantly higher PSS-Fa and QOL-AD scores than the continuity and conventional groups, with the scores of the continuity group being significantly higher than those of the conventional group (*p* < 0.05, Table 5).

**Table 5.** **Comparison of PSS-Fa and QOL-AD scores before and 3 months after implementation of care protocols amongst three groups (points)**.

Group	PSS-Fa score		QOL-AD score	
	Before implementation	Three months after implementation	Before implementation	Three months after implementation
Conventional group (n = 42)	4.13 ± 0.65	7.44 ± 0.78*	26.27 ± 2.81	32.39 ± 3.74*
Continuity group (n = 49)	4.18 ± 0.62	8.15 ± 0.81*^a^	26.84 ± 3.02	37.43 ± 3.95*^a^
Family group (n = 44)	4.09 ± 0.64	9.56 ± 0.83*^ab^	27.11 ± 3.19	41.58 ± 4.16*^ab^
*F*	0.234	77.426	0.868	58.075
*p*	0.792	<0.001	0.422	<0.001

Note: *p < 0.05 versus before implementation within the same group; ^a^*p* < 0.05 versus conventional group; ^b^*p* < 0.05 versus continuity group. PSS-Fa, Perceived Social Support-Family scale; QOL-AD, Quality of Life in Alzheimer’s Disease scale.

## Discussion

With the accelerating global trend of population aging, the prevalence and mortality rates of AD have risen considerably, establishing it as a major public health concern that severely affects population health and societal development. According to the latest data from the Global Burden of Disease (GBD) 2021 database, AD and other dementias (ADDs) ranked 20th amongst the top 25 level 3 causes of years lived with disability globally [29]. A recent national study based on GBD 2021 data revealed that the annual average increase in the age-standardised incidence rate (ASIR) of ADDs in China was 0.68% (far exceeding the global average of 0.06%), with the number of affected individuals approaching 17 million in 2021—a 322.18% increase compared with that in 1990; furthermore, projections using an autoregressive integrated moving average model for the next 15 years (up to 2036) indicate a persistent and pronounced upward trend in ASIR amongst male and female populations in China [30]. This trajectory indicates that clinical care for AD will face increasingly severe pressures and challenges, underscoring an urgent need to develop and identify more proactive and effective care models and strategies.

The findings of this study demonstrate that at the 3-month assessment, the family group exhibited higher MMSE and MoCA scores than the continuity and conventional Groups. This pattern was consistently observed across all educational substrata (illiterate, primary/junior high school and high school and above), where the family group’s MMSE and MoCA scores were significantly superior to those of the other two groups. These results suggest that the application of continuity of care combined with family supportive care may be associated with improved cognitive function in patients with AD, regardless of their educational background. The underlying reasons for these observations can be analysed as follows: Most family caregivers of patients with AD lack sufficient medical knowledge and nursing skills, limiting their ability to provide effective rehabilitative care. Within the family supportive care protocol, pre-discharge health education for caregivers utilising multimedia resources, such as PowerPoint and short videos, enabled a clear understanding of the importance and specific components of family supportive care. Demonstrations and role-playing exercises facilitated the acquisition of daily care techniques, such as guided feeding, thereby creating a conducive home environment for patient rehabilitation. Daily interactions initiated by caregivers, including conversations, storytelling and reminiscence therapy, help patients revisit past life experiences. This process aids in preserving residual memory and, through memory retrieval and the reconstruction of positive narratives, fosters a constructive self-concept whilst stimulating the memory centres of the brain, thereby reinforcing memory function. These interactive engagements also serve to exercise the patient’s logical thinking and attention, contributing to cognitive improvement. Furthermore, the implementation of three scheduled 15 min of daily music sessions, during which patients were guided to perform simple motor activities like clapping or stepping in rhythm with the music, creates a comfortable atmosphere. Soothing music can alleviate patient tension and anxiety, and maintaining a pleasant emotional state promotes a more active brain condition, thereby enhancing cerebral coordination, reactivity and ultimately, cognitive function [31]. Within the continuity of care protocol, individualised plans encompassing dietary management, language function training and motor exercises were formulated before discharge. The combination of WeChat video follow-ups and home visits allowed for the monitoring of patient progress and the provision of tailored guidance and adjustments. This approach stimulates brain function and enhances cognitive capacity through the repetitive reinforcement of daily activities [32, 33]. From a physiological perspective, physical exercise promotes blood circulation, ensuring the brain receives ample oxygen and essential nutrients, thus creating a favourable environment for optimal neuronal function. The integrated care model incorporated a structured exercise regimen, including balance and coordination training. These activities require patients to maintain focus and plan movement sequences, thereby implicitly training cognitive domains, such as attention, spatial perception and executive function, leading to cognitive enhancement [34, 35].

Arman *et al*. [36] demonstrated that the application of family support interventions in patients with myocardial infarction improved their self-care behaviours. A randomised controlled trial conducted by Diriba *et al*. [37] involving Ethiopian patients with type 2 diabetes found that family supportive care enhanced patients’ self-care ability and optimised glycaemic and lipid control levels. A qualitative meta-analysis by Schulman-Green *et al*. [38] indicated that family supportive care contributes to improved self-management capabilities in patients with chronic, disabling diseases. The results of the present study, showing that the family Group exhibited higher BI, PSS-Fa and QOL-AD scores than the continuity and conventional groups at the 3-month assessment, are consistent with the conclusions of the aforementioned studies. This results suggest that continuity of care combined with family supportive care may be associated with enhanced self-care ability, improved family support and increased quality of life in patients with AD. This synergistic effect may be attributed to the following mechanisms: Continuity of care reinforces and consolidates training content through ongoing follow-up, promoting the patient’s mastery of various self-care skills. Complementarily, the family supportive care protocol included structured training plans for ADL. By applying learned techniques, caregivers actively encouraged and guided patients to independently perform tasks such as dressing, tooth brushing and face washing. When resistance was encountered, caregivers utilised acquired strategies to manage the situation effectively. This approach fostered patients’ self-identity and confidence, encouraging active participation in ADL training and ensuring the effective implementation of self-care ability exercises [39]. Within the family supportive care framework, caregivers focused on maintaining patients’ personal hygiene, which helps preserve their sense of dignity. As patients demonstrated progress in cognitive, motor and daily living skills, caregivers provided timely praise and encouragement. These positive interactions within the family setting allowed patients to feel recognised and valued, ameliorating negative emotional states such as anxiety. Consequently, patients experienced greater comfort and satisfaction, leading to an enhanced quality of life. The combination of continuity of care and family supportive care provided a comprehensive rehabilitation plan. Furthermore, the establishment of a dedicated WeChat communication group for caregivers facilitated the sharing of challenges, experiences and insights. This platform enabled caregivers to receive timely professional advice from healthcare staff and learn practical caregiving techniques from peers, thereby alleviating caregiving burden, strengthening their confidence and competence and ultimately enabling the provision of more stable and reliable family support for the patients.

Limitations and future research directions: Firstly, the retrospective nature of the study meant the sample size was constrained by available historical data, precluding a priori sample size estimation, which may have resulted in insufficient statistical power. Future studies should calculate the sample size prospectively on the basis of estimated effect sizes and desired statistical power to ensure adequate study robustness. Secondly, the collection of baseline data may not have fully accounted for residual confounding from unmeasured or under-reported variables. Key confounders related to AD outcomes, such as socioeconomic status and specific caregiver-related factors, may have been omitted, making it difficult to rule out the possibility that the observed effects were attributable to these confounding factors rather than the care protocols themselves. Furthermore, the study did not employ propensity score matching (PSM) to control for potential confounders, allowing baseline differences to potentially influence the accuracy of the results and weaken the persuasiveness of the conclusions. Future research should adopt a prospective cohort design with randomisation by using a random number table to ensure simultaneous initiation and uniform duration of the protocols across groups, thereby controlling for time-related confounding. The concurrent application of PSM is recommended to enhance baseline comparability. Thirdly, the absence of a natural disease progression (observational) control group is a considerable limitation. Given that AD is a progressive disorder characterised by a natural decline in cognitive function and self-care ability, the current findings cannot definitively determine whether the care protocols achieved ‘true improvement’ or merely ‘delayed decline.’ Future studies should include a natural progression control group to further clarify the effects of the protocols. Fourthly, the lack of subgroup analyses based on individual patient characteristics prevents the identification of specific AD subpopulations for whom the care protocols may be most precisely applicable. Particularly within the educational substrata, the sample sizes were limited (e.g., only 8–10 patients per group in the high school and above stratum), resulting in low statistical power for these analyses and a potential risk of type II errors (false negatives). Future investigations should perform subgroup analyses considering individual differences and ensure adequate sample sizes within subgroups to increase statistical power. Fifthly, the outcome measures of this study relied on subjective scales, which may be susceptible to assessor and recall biases. Subsequent research should integrate subjective scales with objective indicators. The introduction of objective blood-based biomarkers, such as phosphorylated Tau protein 217 (p-tau217) and the β-amyloid 42/40 ratio (Aβ42/Aβ40), is recommended. Compared with cerebrospinal fluid biomarkers, which require lumbar puncture for sample collection, and MRI neuroimaging indices, which involve high costs and relatively complex procedures, p-tau217 and Aβ42/Aβ40 samples can be obtained via routine venipuncture. This approach offers considerable advantages, including minimal invasiveness, convenient detection workflows, ease of sample storage and transportation and relatively low cost. These characteristics better align with the practical requirements for clinical dissemination and application, facilitating a more objective and precise evaluation of the effects of the care protocols and reducing the effect of subjective bias. Sixthly, the relatively short follow-up period of 3 months precludes evaluation of the long-term sustainability of the effects. It cannot verify whether the protocols delay progression to moderate or severe disease stages (e.g., GDS stage 4) or influence long-term caregiver burden. Short-term score improvements may be influenced by initially high caregiver adherence, and long-term effects could diminish due to disease progression or waning adherence. Future work should involve long-term follow-up assessments extending for 1 year or more to evaluate the effect of the protocols on disease progression, rates of institutional care dependency and long-term caregiver burden. Seventhly, the study did not analyse potential interactions amongst patient medication adherence, specific drug types and the effects of the care protocols, making it difficult to isolate the ‘pure’ effect of the non-pharmacological components. Future research could employ multiple linear regression models, incorporating variables, such as care group assignment, medication adherence rates and types of medications used, to further explore the synergistic or independent contributions of medication adherence, drug type and the care protocols on the outcome measures. Eighthly, the study did not evaluate the differences in human resource, time and financial costs associated with the three care models. This information is crucial for assessing the feasibility and scalability of the protocols in real-world settings. Future studies could explore optimised, lower-cost implementation strategies, such as streamlining training content or utilising centralised online training platforms, to enhance the accessibility and sustainability of these care models.

## Conclusions

The findings of this retrospective analysis indicate that the integrated application of continuity of care and family supportive care in patients with AD is associated with positive effects on cognitive function, self-care ability, family support and quality of life. These results suggest that this combined care model may represent a superior alternative for the management of AD. However, given the inherent limitations of retrospective designs in fully controlling for confounding variables, further validation through prospective, large-scale studies is warranted to confirm its efficacy and establish its potential for broader implementation.

## Availability of Data and Materials

The original contributions presented in the study are included in the article. Further inquiries can be directed to the corresponding author.
